# Relativistic correction of atomic scattering factors for high-energy electron diffraction

**DOI:** 10.1107/S2053273319012191

**Published:** 2019-10-24

**Authors:** Markus Lentzen

**Affiliations:** a Ernst Ruska Centre, Forschungszentrum Jülich GmbH, 52425 Jülich, Germany

**Keywords:** electron diffraction, atomic scattering factors, relativity theory, Schrödinger equation

## Abstract

Relativistic electron diffraction depends on linear and quadratic terms in the electric potential, the latter being neglected in the frequently used relativistically corrected Schrödinger equation. The quadratic electric potential term modifies atomic scattering amplitudes in particular for large-angle scattering and backscattering. The respective correction increases with increasing scattering angle, increasing atomic number and increasing kinetic energy. Conventional tabulations for electron scattering and its large-angle extrapolations can be amended in closed form by a universal correction based on the screened Coulomb potential squared.

## Abstract 2

Relativistic electron diffraction depends on linear and quadratic terms in the electric potential, the latter being neglected in the frequently used relativistically corrected Schrödinger equation. Conventional tabulations for electron scattering and its large-angle extrapolations can be amended in closed form by a universal correction based on the screened Coulomb potential squared.

## 1. Introduction   

A frequently used framework for the calculation of high-energy electron diffraction by an atom or ion is the solution of the relativistically corrected Schrödinger equation (Molière, 1947 ▸; Fujiwara, 1961 ▸) with a model for the atomic or ionic electric potential. These model potentials are tabulated for a wide range of atomic numbers and frequently occurring ionic charges in the form of scattering factors (Doyle & Turner, 1968 ▸; Doyle & Cowley, 1974 ▸; Rez *et al.*, 1994 ▸, 1997 ▸) or their parameterizations (Doyle & Turner, 1968 ▸; Doyle & Cowley, 1974 ▸; Fox *et al.*, 1989 ▸; Rez *et al.*, 1994 ▸, 1997 ▸; Waasmaier & Kirfel, 1995 ▸; Weickenmeier & Kohl, 1998 ▸; Peng, 1998 ▸; Lobato & Van Dyck, 2014 ▸); see Kirkland (2010 ▸) for a survey. Conventionally, tables of the scattering factors display the Born scattering amplitude, that is the Fourier transform of the electric potential times an interaction constant. A relativistic correction, dependent on the electron speed, is applied to the tabulated values, which can be directly used to determine scattering cross sections on the first Born approximation.

The normal form of the relativistically corrected Schrödinger equation (Molière, 1947 ▸; Fujiwara, 1961 ▸) is linear in the electric potential, yet the correct relativistic energy-momentum relation, which is the basis of the Klein–Gordon equation (Klein, 1926 ▸; Gordon, 1926 ▸; Kragh, 1984 ▸), contains an additional quadratic term in the electric potential. That term is neglected in the above conventional framework, and thus, to the best of our knowledge, no tabulations exist for fully corrected relativistic scattering factors.

The aim of this work is to explore the impact of the quadratic electric potential term on atomic or ionic electron scattering amplitudes in particular at large angles, including backscattering. Furthermore, a method is proposed to amend the existing tables for the Born scattering factors. The work presents a brief survey of the required theory, calculations for a set of atoms of small, medium and large atomic number at small, medium and large electron energy, and concludes with a discussion of possible applications.

## 2. Theory   

### 2.1. Wave equations   

The relativistic energy-momentum relation (Einstein, 1905 ▸) 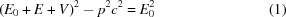
with rest energy 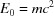, kinetic energy *E* in vacuum, potential energy 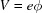, momentum *p*, speed of light *c*, rest mass *m*, elementary charge *e* and electric potential 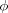 is divided by 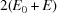 and rearranged: 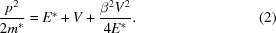
Thus the quadratic energy relation (1) adopts a form akin to a linear energy relation with the parameters 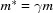, 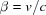, electron speed *v*, 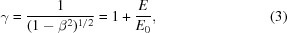
and 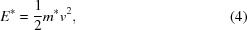
the relativistically modified kinetic energy in vacuum.

The Klein–Gordon equation (Klein, 1926 ▸; Gordon, 1926 ▸; Kragh, 1984 ▸) for fixed kinetic energy is derived by substituting the momentum operator 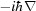 for the momentum *p*, and the relativistically corrected Schrödinger equation (Molière, 1947 ▸; Fujiwara, 1961 ▸) by further neglecting the squared potential term. As usual, 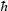 denotes the Planck constant *h* divided by 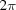.

The scattering amplitude 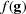 is derived from the wave equations by an *ansatz* for the wavefunction, 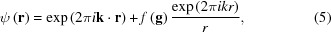
which describes the scattering of a plane wave with wavevector 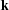 into a spherical wave with an amplitude dependent on the scattering vector 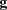. The wavevector of a plane partial wave after scattering is thus 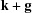, and 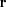 denotes a coordinate in real space. For elastic scattering 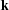 and 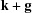 are equal in magnitude, 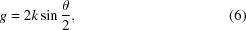
and 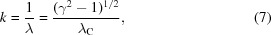
with 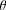 the scattering angle between 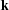 and 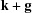, and 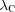 the Compton wavelength.

The amplitude of the spherical wave is determined in the far field, at large distance *r* from a scattering region bounded by a sphere of diameter *d*, with 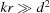. If the bounded region contains a single atom, the scattering amplitude is called the atomic form factor. The far-field solution of the wave equation in the above sense is found on the first Born approximation (Born, 1926 ▸) to the first order of an effective potential 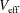, with the well-known result:
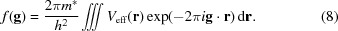
For the relativistically corrected Schrödinger equation 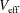 = 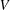, and for the Klein–Gordon equation 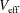 = 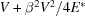. The tabulations of atomic form factors according to equation (8) are used in two ways. The first, and obvious, is the display of the atomic scattering amplitude, its modulus squared being the differential scattering cross section (see the next section). The second is the indirect, through the Fourier transform in equation (8), but exact display of the atomic scattering potential.

### 2.2. Scattering amplitudes for a screened Coulomb potential   

The integral (8) for the scattering amplitude of a screened atomic Coulomb potential (Wentzel, 1926 ▸) 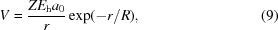
with atomic number *Z*, Hartree energy 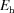, Bohr radius 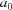 and screening radius (Lenz, 1954 ▸)
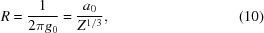
can be solved in closed form, with the well-known result (Wentzel, 1926 ▸): 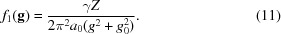
The scattering amplitude of the squared atomic Coulomb potential term 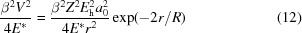
can be found in closed form as well: 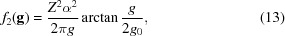
with the fine structure constant 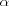.

Both 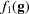 and 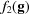 have a maximum at 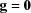, 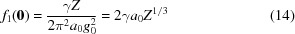
and 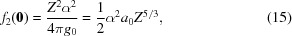
and for small scattering vectors 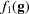 is always much larger than 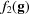. A comparison of the asymptotes for large scattering vectors, 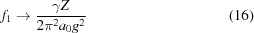
(Rutherford, 1911 ▸) and 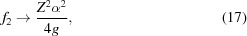
reveals that 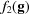 can become equal in magnitude to 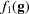 for large *Z*. This is particularly clear for the asymptotic values of backscattering 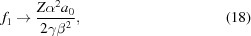
and 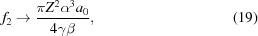
when 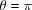 and *g* adopts the largest possible value 2*k*. The contribution 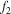 to the total scattering amplitude 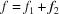 becomes significant for large-angle scattering and backscattering.

The differential cross section is 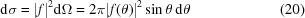
for scattering into a solid angle 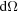 and azimuthal symmetry.

## 3. Calculation of scattering amplitudes and cross sections   

**Figure 1:**
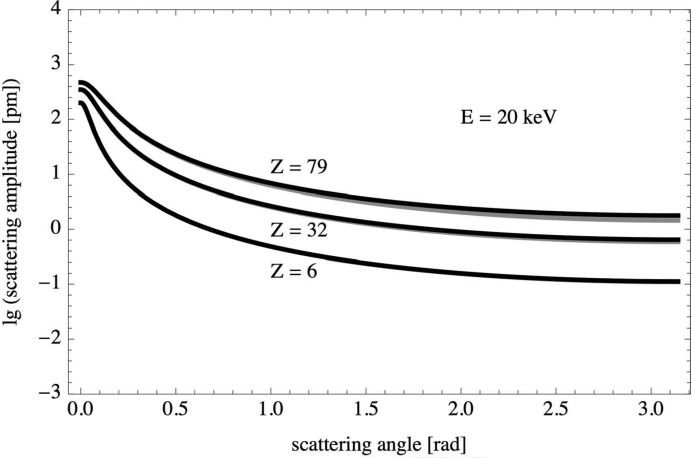
Born scattering amplitudes 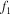 (grey) and 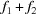 (black) on a logarithmic scale versus scattering angle for carbon, germanium and gold at a kinetic energy of 20 keV.

Born scattering amplitudes [equation (8)] were calculated for carbon (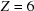), germanium (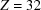) and gold (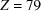) at kinetic energies of 20, 200 and 2000 keV over the full range of scattering angles, 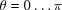. Two different models were used for the scattering potential: the screened Coulomb potential [equation (9)] and the screened Coulomb potential extended by the squared Coulomb potential term [equation (12)]. The scattering amplitudes for both models, 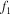 and 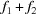, are displayed in Figs. 1 ▸, 2 ▸ and 3 ▸. The difference between the two scattering amplitudes increases with increasing scattering angle, increasing atomic number and increasing kinetic energy.

The difference between the two models can be further expressed by calculating Born scattering cross sections for total scattering, 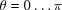, which denotes the total mismatch. The relative difference between the two models is pronounced for large scattering angles, and thus it is instructive to further calculate the cross section for backscattering, 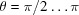. A third important measure is the cross section for scattering outside the typical acceptance angle of an electron microscope, θ = 250 mrad…π.

The respective cross sections on the two models, and the relative differences, are compiled in Tables 1 ▸, 2 ▸ and 3 ▸, again for carbon (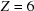), germanium (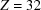) and gold (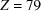) at kinetic energies of 20, 200 and 2000 keV. The relative differences of the total cross sections decrease for increasing energy; they increase for backscattering and scattering outside the microscope acceptance angle with increasing energy. With increasing atomic number the relative differences increase in any category.

## 4. Discussion   

Conventional tables of the scattering factors 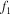 (Doyle & Turner, 1968 ▸; Doyle & Cowley, 1974 ▸; Rez *et al.*, 1994 ▸, 1997 ▸; Kirkland, 2010 ▸) are organized such that the Born scattering amplitude [equation (8)] is only tabulated for a range of scattering vectors where Rutherford scattering is modified by the effects of screening, up to, *e.g.*, *s* = *g*/2 = 60.0 nm^−1^. The amplitudes for larger scattering vectors are understood to be calculated with the Rutherford formula [equation (16)]. In a last step the tabulated values have to be multiplied by γ as the interaction constant used in the tabulations conventionally contains *m* and not 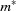.

The above standard procedure can be amended to include the effects of the squared potential term [equation (12)], thus providing a proper relativistic correction. Although 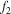 was calculated for the screened Coulomb potential in equation (13), it can serve as a universal correction, because in the range of small scattering vectors, where the details of the screening would play a role, 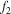 is dominated by 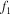. For larger scattering vectors, in the regime of Rutherford scattering [equation (16)], 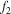 has the proper asymptote [equation (17)].

Thus the conventional tables can be used to derive the proper relativistic scattering amplitude:

(i) Multiply tabulated values by γ.

(ii) Extrapolate the tabulated range through the Rutherford formula [equation (16)].

(iii) Determine the screening parameter 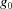 using equation (10).

(iv) Add the squared potential term 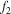 using equation (13).

Once the scattering amplitude is determined, a Fourier transform to real space provides the effective potential to be used in diffraction calculations on the Klein–Gordon equation. The above treatment of the squared potential term allows, however, the use of simpler algorithms for the solution of the Schrödinger equation instead.

The implementation of the proper relativistic scattering amplitude is particularly suitable for the phase grating approximation of the multislice algorithm (Cowley & Moodie, 1957 ▸). The calculation of the phase grating requires a projection of the potential along the chief propagation direction, which is achieved by evaluating the structure factor of an atomic arrangement with the component of the scattering vector along the propagation direction set to zero. Including the squared potential term would now involve a Fourier transform to real space, calculation of the squared potential and line integrations along the propagation direction, or alternatively a numerically costly convolution in reciprocal space. Compared with the latter, the prescription given in this work provides a numerically very efficient way to determine the respective additional structure factor based on the form factors 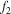.

The squared potential correction [equation (13)] is obviously most significant for backscattering, as can be deduced from the scattering cross sections displayed in the rightmost columns of Tables 1 ▸, 2 ▸ and 3 ▸. The error by neglecting the correction can be as large as 66.7% for the case of gold at a kinetic energy of 2 MeV. The modification of backscattering cross sections extends, however, into the region of medium electron energies and medium to small charge numbers. A striking example is the cross section for knock-on damage in germanium, which involves scattering angles from 2.5 rad to π for a kinetic energy of 400 keV to transfer the required displacement energy of 15 eV to a germanium atom. The Born cross section for this process is 0.00316 pm^2^, but only 0.00189 pm^2^ by neglecting correction (13), which is a difference of 40.2%. An example of knock-on damage of a light element is oxygen displacement in magnesium oxide at a displacement energy of 55 eV. The Born cross section for this process at 400 keV electron energy is 0.000504 pm^2^, but only 0.000441 pm^2^ by neglecting correction (13), which is a difference of 12.5%.

The modification of the cross sections for scattering outside the acceptance angle of an electron microscope indicates that there is also an impact on an important parameter of forward scattering, namely electron absorption. In transmission electron microscopy the bore of the objective pole-piece limits the cone of scattered electrons to a semi-angle of around 250 mrad, and thus a certain fraction of scattered intensity is missing in the image plane underneath; it appears to be absorbed by the imaging system. For larger kinetic energies and larger atomic numbers the estimate of that apparent electron absorption would be in error on the linear model 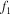 alone.

## 5. Conclusion   

The conventional framework of electron scattering by an electric potential is modified by an additional quadratic term in the electric potential, if the correct relativistic energy-momentum relation (1) is considered. The respective modification of atomic scattering amplitudes increases with increasing scattering angle, increasing atomic number and increasing kinetic energy. Conventional tabulations for electron scattering (Doyle & Turner, 1968 ▸; Doyle & Cowley, 1974 ▸; Rez *et al.*, 1994 ▸, 1997 ▸; Kirkland, 2010 ▸) and its large-angle extrapolations can be amended in closed form by a universal correction [equation (13)] based on the screened Coulomb potential squared [equation (12)].

## Figures and Tables

**Figure 2:**
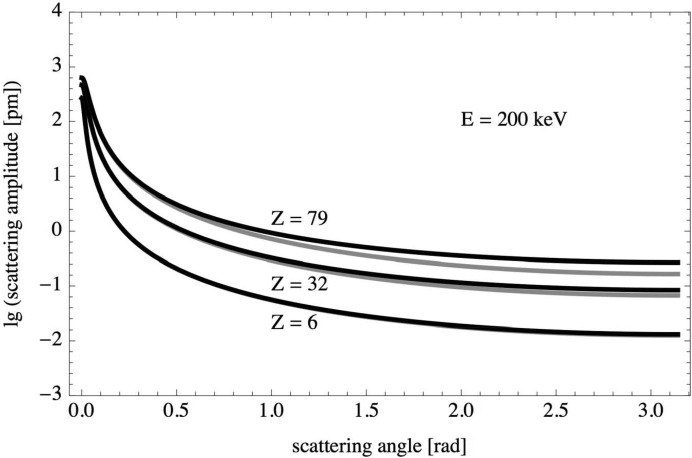
Born scattering amplitudes 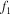 (grey) and 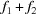 (black) on a logarithmic scale versus scattering angle for carbon, germanium and gold at a kinetic energy of 200 keV.

**Figure 3:**
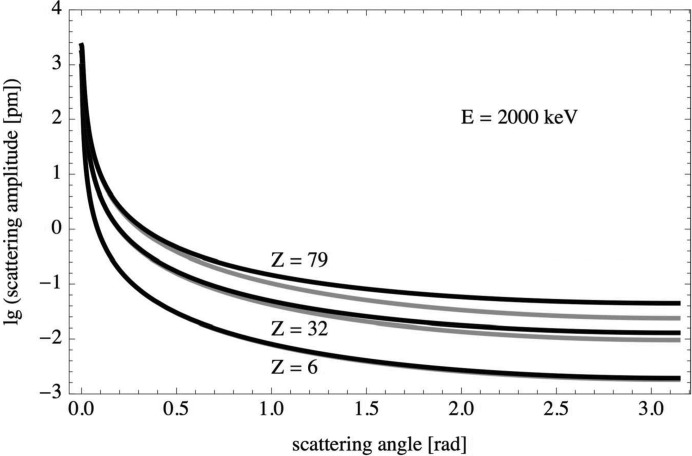
Born scattering amplitudes 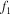 (grey) and 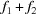 (black) on a logarithmic scale versus scattering angle for carbon, germanium and gold at a kinetic energy of 2000 keV.

**Table 1:** Born scattering cross sections σ (pm^2^) and relative errors for carbon, for various scattering angles and kinetic energies

θ	Total	> 250 mrad	> π/2
20 keV			
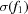	276.3	9.31	0.152
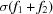	276.5	9.38	0.157
	0.10%	0.73%	2.92%
200 keV			
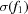	42.26	0.125	0.00198
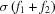	42.29	0.128	0.00214
	0.07%	2.01%	7.40%
2000 keV			
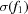	21.31	0.00256	0.0000405
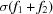	21.32	0.00264	0.0000451
	0.02%	2.90%	10.3%

**Table 2:** Born scattering cross sections σ (pm^2^) and relative errors for germanium, for various scattering angles and kinetic energies

θ	Total	> 250 mrad	> π/2
20 keV			
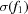	2571.4	247.1	4.31
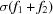	2593.9	256.3	5.00
	0.87%	3.61%	13.9%
200 keV			
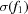	393.7	3.54	0.0564
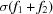	396.4	3.93	0.0823
	0.68%	9.90%	31.4%
2000 keV			
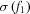	198.6	0.0729	0.00115
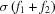	199.0	0.0849	0.00194
	0.20%	14.2%	40.5%

**Table 3:** Born scattering cross sections σ (pm^2^) and relative errors for gold, for various scattering angles and kinetic energies

θ	Total	> 250 mrad	> π/2
20 keV			
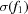	8567.8	1391.6	26.15
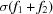	8815.1	1518.4	36.89
	2.80%	8.35%	29.1%
200 keV			
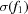	1313.5	21.42	0.344
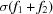	1343.9	27.41	0.784
	2.26%	21.8%	56.1%
2000 keV			
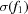	662.6	0.444	0.00702
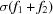	667.0	0.638	0.02106
	0.66%	30.4%	66.7%
